# Recommendations for head and neck surgical procedures during the COVID-19 pandemic

**DOI:** 10.6061/clinics/2020/e2084

**Published:** 2020-07-02

**Authors:** Marco A.V. Kulcsar, Fabio L.M. Montenegro, André B.O. Santos, Marcos R. Tavares, Sergio S. Arap, Luiz P. Kowalski

**Affiliations:** I Servico de Cirurgia de Cabeca e Pescoco, Hospital das Clinicas HCFMUSP, Faculdade de Medicina, Universidade de Sao Paulo, Sao Paulo, SP, BR; II Servico de Cirurgia de Cabeca e Pescoco, Instituto do Cancer do Estado de Sao Paulo (ICESP), Hospital das Clinicas HCFMUSP, Faculdade de Medicina, Universidade de Sao Paulo, Sao Paulo, SP, BR

**Keywords:** COVID-19, Surgery, Head and Neck, Prevention, Complications

## Abstract

The coronavirus disease (COVID-19) pandemic, caused by severe acute respiratory syndrome coronavirus 2 (SARS-CoV-2), has spread exponentially worldwide. In Brazil, the number of infected people diagnosed has been increasing and, as in other countries, it has been associated with a high risk of contamination in healthcare teams. For healthcare professionals, the full use of personal protective equipment (PPE) is mandatory, such as wearing surgical or filtering facepiece class 2 (FFP2) masks, waterproof aprons, gloves, and goggles, in addition to training in care processes. A reduction in the number of face-to-face visits and non-essential elective procedures is also recommended. However, surgery should not be postponed in the case of the most essential elective indications (mostly associated with head and neck cancers). As malignant tumors of the head and neck are clinically time sensitive, neither consultations for these tumors nor their treatment should be postponed. Postponing surgical treatment can result in a change in the disease stage and alter an individual's chance of survival. In this situation, planning of all treatments must begin with the request for, in addition to routine examinations, a nasal swab polymerase chain reaction for SARS-CoV-2 and chest computed tomography. Only if the results of these tests are positive or if fever or other symptoms suggestive of COVID-19 are present should the surgical procedure be postponed until the patient completely recovers. This is mandatory not only because of the risk of contamination of the surgical team but also because of the increased risk of postoperative complications and high risk of death. During this pandemic, the most effective safety measures are social distancing for the general public and the adequate availability and use of PPE in the healthcare field. The treatment of other chronic diseases, such as cancer, should be continued, as the damming of cases of these diseases will have a deleterious effect on the public healthcare system.

## INTRODUCTION

COVID-19 is a systemic infectious disease caused by severe acute respiratory syndrome coronavirus 2 (SARS-CoV-2), which compromises several systems of the body. It started in Wuhan (Hubei province), China, on December 31, 2019. With the rapid spread of the epidemic, an international health emergency was declared by the WHO on January 30, 2020 (1,2). The first case in Brazil was registered on February 26, 2020 (3). In less than 3 months, this disease had spread to the entire national territory, and by May 25, 2020, 374,898 cases and 23,473 deaths had been officially registered (4).

SARS-CoV-2 is an RNA virus that is present in the upper and lower airways and fecal matter. Contamination generally occurs by the transmission of the virus through saliva droplets or through aerosols generated when speaking, sneezing, or coughing. These can be dispersed up to 2 meters, although there is a possibility that it can reach an even greater distance (5). The virus remains in suspension, in the external environment, for a brief period, but can remain alive on surfaces, such as plastic, for up to 72 hours (6).

According to the Robert Koch Institute, which monitors infectious diseases in Germany, it is estimated that the basic reproduction rate (R) of COVID-19 is between 2.4 and 3.3 (7); that is, an infected individual can spread the disease to 2 to 3 other people (8). Contagion is caused by contact of the virus with the oral, nasal, or ocular mucosae. It has been documented that humans can infect domestic animals, but it is not known whether these animals can transmit the virus to humans (9).

The incubation period varies from 2 to 15 days, with an average onset of symptoms at 5 to 6 days. The most frequent symptoms are fever, fatigue, myalgia, headache, and dyspnea. Dyspnea is associated with the most severe cases, which represent about 20% of those infected, with around 5% mortality in this group (10). Cases with anosmia, hyposmia, and ageusia (11); gastrointestinal conditions with diarrhea (12); and painful abdominal lymphadenopathy (13) have also been identified. The diagnosis is made using clinical symptoms, nasal swab reverse transcriptase-polymerase chain reaction (RT-PCR) for SARS-CoV-2 (gold standard for diagnosis in the acute phase), IgG and IgM serology (later stage), and chest computed tomography (especially in patients with respiratory symptoms). This virus does not yet have a specific treatment and the recommended measures to reduce the risk of contagion are social distancing and the use of masks (14,15).

### Risk for healthcare professionals

As the transmission of SARS-CoV-2 between humans occurs through respiratory secretions, healthcare professionals who work in emergency care or in manipulation of the oral and maxillofacial region are more exposed to the risk of contamination. Healthcare professionals who worked with infected patients in the city of Wuhan (China) accounted for 3.8% of cases; of those, 14.8% had a severe clinical presentation and 0.6% died (16).

The hospital environment was responsible for 41% of coronavirus transmission in China, both for healthcare teams and for patients. The same occurred in Italy (17). In Brazil, on May 14, 2020, it was reported that 31,700 healthcare professionals were infected with COVID-19 (18). When infected, these professionals must be temporarily removed from their duties and, in most cases, their replacement is difficult, as there is no way to quickly train substitutes (19-22). Unfortunately, some of these professionals develop severe forms of the disease and can die. In Brazil, the Federal Nursing Council reported the deaths from COVID-19 of 108 nursing professionals up to May 13, 2020 (23).

As mentioned above, because of the nature of their work, specialists in head and neck surgery (HNS) and otorhinolaryngology (ENT); dentists; oral and maxillofacial surgeons; cranio-maxillofacial surgeons; thoracic surgeons; endoscopists; pulmonologists; ophthalmologists; neurosurgeons; physiotherapists; nurses; and speech therapists have greater exposure to the virus because of direct contact with secretions from the upper respiratory tract. This greater exposure occurs during locoregional clinical evaluation, including examination or fibroscopy of the nose, oral cavity, or pharynx, and during treatment, especially when a surgical intervention is performed. The SARS-CoV-2 virus is highly concentrated in the nasal, oral, and oropharyngeal mucosae. Thus, any manipulation of this region can generate aerosols and promote contagion of the professional (21,24-30).

### Protection for healthcare professionals

The best ways to reduce the risk of infection in healthcare professionals are to employ measures to reduce contagion and provide multidisciplinary training. In this regard, individual protection recommendations have been made by several national and international medical societies, which can be adapted to our context, as described below (21,22,24-29,31-37).

During the epidemic, regardless of the presence of clinical signs and symptoms, appropriate personal protective equipment (PPE) should be used, and an appropriate location should be chosen for clinical, endoscopic, and surgical procedures. These precautions must be taken by the healthcare professionals who participate in these procedures. There is also an obligation for health authorities at various levels to provide material resources and training, so that the healthcare system is not exhausted because of contamination of professionals working to combat the epidemic. This issue has led to the deaths of healthcare professionals since the beginning of the COVID-19 pandemic, and unfortunately it continues to occur, mainly because of a lack of adequate PPE (20,21,24,26,27,32) or, where PPE is adequately provided, failure to train in proper donning and removal (38).

In addition, to minimize the risk of contamination of healthcare professionals during the pandemic, consultations and elective surgeries should be avoided, especially in a scenario with a significant increase in the number of infected individuals. It is important to note that there are numerous asymptomatic individuals in the preclinical phase who nevertheless have transmission capacity (4,23). However, there are patients with diseases that progress, for which treatment cannot be postponed. Furthermore, it is not known for how long the pandemic situation will last. The rapid control obtained in some countries in the East has not been replicated in other parts of the world. Thus, consultations and procedures should be carried out according to the severity of the disease.

### Consultations and diagnostic procedures

Despite the high risk of contamination by SARS-CoV-2 during the pandemic, there are chronic diseases that require clinical follow-up to avoid complications or, in some cases, even death; for example: rheumatic, metabolic, and cardiovascular diseases and cancer. In this situation, for stable cases, a good alternative is to use telemedicine for doctor-patient communication, with a record in the medical chart according to the regulations of the Brazilian Federal Council of Medicine (FCM) (30). In case of the need for a face-to-face consultation, the risk of the patient having an asymptomatic infection should be considered. The patient must wear a surgical mask and the doctor must provide care while wearing full PPE (FFP2 protective mask, gloves, waterproof apron, and goggles or face shield) in an appropriate place with good ventilation (20,21,24-27,29,32-35,37).

During the pandemic, all patients should be considered as possible disease carriers during elective or emergency care, not only those with symptoms of upper airway infection. Full PPE must always be adopted, the examination room must be well ventilated, only a highly experienced doctor should be present in the room, and, when possible, care should be provided without an assistant (24,26,27,29,37).

A complete evaluation requires examination with endoscopes (high risk of contamination). Nasofibrolaryngoscopies cause dispersion of viral particles in an aerosolized environment. For this reason, during the pandemic, this examination should be restricted to those with a clinical history and symptoms of suspected malignancy (dysphonia, dysphagia, cervical mass, cancer risk population) or with neck infections. It should be performed in a separate room from that used for routine care, with a ventilated environment and the air conditioning off, but the ideal location to perform the examination is a negative pressure room, if possible. The doctor must be highly experienced in conducting the examination and must be donned with complete PPE, and the presence of other people in the room is not recommended. For the examination, the use of adequate topical anesthesia is suggested, to avoid the coughing or vomiting reflex. After the exam, standard removal of PPE must be performed, the environment must be cleaned, and the endoscopy material must be sent for proper sterilization (4,21,24,26,27,29,30,37). It is important that the institution's guidelines are followed by the examiner. If the institution has not advised on the use of materials and their disposal, the physician must report this to the Hospital Infection Commission.

It is clear that training of the healthcare team is essential to ensure the proper use of PPE. PPE should be used during procedures with a risk of contamination, which were previously performed without proper protection in an outpatient environment, such as: the routine aspiration of patients with tracheostomy or cannula replacement; placement, removal, or manipulation of nasal catheter; or exchange of tracheoesophageal prosthesis. Some procedures previously performed on an outpatient basis should currently be conducted in an operating room, such as: drainage of cervical and peritonsillar abscesses and removal of foreign bodies (29,31,37). These changes should be incorporated into the regular routine, even for a few months after the pandemic.

### Indications for head and neck cancer surgery

At the beginning of the pandemic, elective surgeries were postponed, guided by the World Health Organization and endorsed by most national and international medical societies, as the pandemic necessitated an increase in the consumption of hospital resources, such as PPE, and a relocation of medical teams (26,30,39,40).

Thus, there is a need for an eligibility classification, such as that by Stahel described below, that incorporates new classes such as elective urgency surgery (cases of malignant head and neck tumors) and essential elective surgery (represented by other diseases of the specialty) (41):

- Emergency – surgery within 1 hour – examples: exploratory cervicotomy for bleeding and/or hematoma or tracheostomy for acute obstructive respiratory failure;
- Urgency – surgery within 24 hours – example: infection of the neck with abscess and airway compression;
- Elective urgency - surgery within 2 weeks – examples: operable tumors of the oral cavity, pharynx, or larynx, with bleeding, severe pain, or mild obstructive signs, or compressive goiter with obstructive symptoms or hyperparathyroidism refractory to drug treatment;
- Essential elective – surgery within 1 to 3 months – examples: malignant tumors of the upper aerodigestive tract or salivary glands, skin cancer with metastasis or close to noble structures, sarcomas, or thyroid carcinomas (anaplastic, medullary, poorly differentiated, and papillary, with metastasis or invasion of central compartment structures);
- Non-essential elective - surgery after 3 months or more – examples: goiter, hyperparathyroidism, or benign diseases of the cervicofacial segment.

However, even these classifications require adjustments. Clinically necessary and time-sensitive procedures are often not supported by medical and hospital resources during an epidemic. Thus, to take a more objective and ethical approach, especially in cases of cancer, the ideal is to adopt a scoring system, rather than just an algorithm based on tests for SARS-CoV-2 or another method. To this end, the scoring system proposed by Prachand et al. (42) is based on the analysis of surgical factors (length of operation, length of stay, need for an intensive care unit in the postoperative period, number of doctors in the surgical team, probability of orotracheal intubation (OTI), and surgical site), disease factors (effectiveness of non-surgical treatment, impact of the postponement of surgical treatment for 2 or 6 weeks on healing outcomes and surgical risk), and patient factors (age and comorbidities). The total score ranges between 25 and 105: the higher the score, the lesser the surgery should be encouraged, because of the risks of postoperative complications, the need for supplies and human resources, and the risk of contamination of the patient and team by COVID-19 (42). This scale is valid as long as the PPE is appropriate for each situation.

### Indications for non-cancer surgeries

There are several non-oncological conditions in Head and Neck Surgery. For example, this specialty deals with congenital disorders, diseases of the cervical endocrine glands, paragangliomas, neurofibromas, epidermal cysts, salivary gland cysts and inflammation, and lipomas, among other conditions. Most of these conditions can have their definitive treatment postponed without risk to the patient. The recommendations of the Specialty Societies, National Supplementary Health Agency, and National Health Surveillance Agency in Brazil are that elective surgeries should be postponed (43,44). This includes a large proportion of non-cancer surgeries.

However, some conditions may require urgent evaluation and even surgical procedures. In the Clinical Hospital of the Faculty of Medicine of the University of São Paulo (HCFMUSP), we classified the following conditions as urgencies during the pandemic: bleeding or bruising, airway obstructions, abscesses or infections with risk of sepsis, severe dysphagia due to benign obstructive processes, hypercalcemic emergence (associated with hyperparathyroidism), and bulky or fast-growing goiters.

Cervical masses that show significant growth in a short time can lead to the suspicion of malignant transformation and must be reassessed promptly to make a decision.

Regarding inflammation of the salivary glands, clinical treatment should be the first choice, especially in cases of sialoadenitis after radioiodine therapy for thyroid cancer.

Thyroglossal duct cysts and branchial cysts can develop infections. In general, they respond favorably to antibiotic therapy. In the case of an abscess, drainage will be required.

The thyroid gland can show two main non-oncological changes that may require urgent surgery: compressive goiter with significant exacerbation of respiratory symptoms or hyperthyroidism refractory to usual drug control or with side effects to drugs (granulocytopenia, hepatitis, or allergy) (41).

Patients with hyperparathyroidism should have their treatment postponed, unless they have a hypercalcemic emergency (total calcium above 14 mg/dL) (45). In the event of neurological symptoms with decreased consciousness, cardiac complications, acute pancreatitis, calciphylaxis, or evidence of acute kidney injury, the operation may be performed even in the presence of lower calcium levels (45). In the case of bone pain, the initial approach should be controlling the pain with analgesics.

It is important to inform patients about the risk of SARS-CoV-2 infection during hospitalization for a procedure (46).

### Protection during head and neck surgery

In cases of oncological diseases where the treatment of choice is surgical, a PCR test for SARS-CoV-2 should be performed 1 to 2 days before surgery (depending on the time for the result to be available), in order to have the result preferably before admitting the patient. If the diagnostic test is positive, whether the procedure can be postponed until the patient's clinical condition improves should be evaluated. When surgery is unavoidable or when the test cannot be performed, consider the flow of the procedure and all care as applicable in the case of surgery for an individual with a suspected or confirmed case of the disease. In addition to the clinical evaluation, a computed tomography scan of the chest can be performed 24 hours before the operation; despite the low evidence for its use, it also serves for restaging immediately before the procedure (5,33,34,37). Even for patients classified as low-risk (in 14-day quarantine, without signs of infection, and with a negative test for COVID-19) with indications for surgical treatment, it is suggested to maintain the same precautions as those recommended for high-risk patients (29,34,35,37).

During transport of patients to the operating room and on referral to the recovery unit, a strict routine must be adopted. For the transportation of extubated patients, transfer with a surgical mask is suggested; if oxygen is required, it can be administered using a facial mask over the first mask. However, in the case of intubated patients, transportation must be with a ventilator coupled to a high-efficiency particulate air (HEPA) filter. The use of complete PPE is mandatory for the entire transfer team (37).

Regarding the operating room for operations on patients at high risk or positive for COVID-19, the ideal is to use an environment with negative pressure; in the absence of this room, the air conditioning should be turned off, thus reducing the movement of air in the environment and the possibility of the virus circulating through the room. The entire team must be dressed in complete PPE, aerosols are generated during OTI, which increases the risk of contamination. Thus, coordination with the anesthesia team is essential during both intubation and extubation. In these two moments, it is recommended that all personnel not essential to the procedure leave the room and only return after the airways are protected (31,37,47).

Another source of contamination is aerosols generated with the use of surgical energy devices for hemostasis during the procedure (electric or ultrasonic scalpels), in addition to other devices such as micromotors, microdebriders, and saws. The use of such equipment should be minimized or avoided; if their use is essential, a vacuum cleaner must be kept close to the aerosol source during use, preferably coupled to a HEPA filter, to reduce dispersion (31,37).

It is not possible to create a rigid protocol for surgical indication based only on the level of risk of contamination during surgery. Because, in the head and neck region, we have benign and malignant diseases, and different procedures, as in the segment of the nasal cavity and pharynx, but being areas of high prevalence of the virus, therefore the procedure has a high risk of contamination for the surgeon and staff, in addition to an increased risk of death for the infected patient. Thus, these procedures should be postponed in infected patients or in those with benign diseases. However, in the case of cancer patients, these procedures must be performed, with mandatory donning of complete PPE by all personnel in the operating room (37).

Other examples are thyroidectomies and neck procedures for malignant neoplasms such as lymph node metastasis, which do not expose the mucosal surface and, therefore, have a lower risk of contamination. Even in these cases, with COVID-19 positive patients, appropriate PPE should be used, as all energy devices for hemostasis generate vapors and can cause viral dispersion by aerosols because of the presence of the virus in the bloodstream (37).

### Tracheostomy

Infection with COVID-19 can cause acute respiratory disease and, in some cases, pulmonary insufficiency, requiring prolonged mechanical ventilation (5% to 15% of patients). The duration may vary, but periods longer than 10 days of OTI are frequent (48). Tracheostomy in patients with suspected or confirmed COVID-19 poses complex challenges, as it is the second most aerosol-generating procedure (49). As with airway teams, the creation of trained and qualified tracheostomy teams reduces the risk of contamination (49). In addition, a detailed peri- and postoperative process must be established to reduce the risks to other patients and healthcare professionals (28,33,35).

A 10-day waiting period for a patient receiving invasive ventilation prior to tracheostomy is important for reducing viral infectivity and assessing the outcome of the disease. Another important point is that, in the first periods of pulmonary involvement, the patient can benefit from prone positions, and this position and the need for extreme changes in position can be considered as contraindications for a tracheostomy (49), mainly because of the risk of pressure injuries to the trachea and cervical vessels. In addition, in these early periods, patients experience significant impairment of gas exchange and require positive pressure, with some unable to tolerate the loss of pressure, even temporarily, that a tracheostomy requires (49).

Regarding the cost-effectiveness of performing an early tracheostomy, it has been shown that a patient undergoing an early tracheostomy is cheaper than one who undergoes a tracheostomy at a later time, and even cheaper than a patient who avoids a tracheostomy (50). In Brazil, because of the lack of critical care beds and the need to turn the intensive care unit (ICU) beds, the request for early tracheostomy to accelerate de-intensification of care is a reality. However, because of the prognosis and risk of death of patients with COVID-19, this cost-effectiveness assessment may not be valid in this context, especially when a tracheostomy is indicated in a patient with a low chance of recovery. This is a broad discussion for the field of bioethics.

A tracheostomy team was created at the Central Institute of the HCFMUSP to concentrate on these procedures and standardize care. It consisted of surgeons from the general surgery, thoracic surgery, head and neck surgery, and otorhinolaryngology departments. This team developed general guidelines, such as the need for multidisciplinary assessment of cases with indications (by an intensivist and two tracheostomy team members). Although it is recognized that before the pandemic we performed early tracheostomy in patients with prolonged intubation to avoid tracheal stenosis in critically ill patients (51), there is much discussion in the literature about the ideal time to perform tracheostomy in patients with COVID-19, as the mortality rate in SARS patients ranged from 15% to 40% of cases undergoing prolonged OTI.

Tracheostomy teams perform daytime procedures on weekdays to ensure that support and back-up teams are available. The technique used is that chosen by each team, in which they are most experienced. The procedure can be open or percutaneous, guided by ultrasonography, avoiding tracheoscopy, and only transporting the patient to the surgical center if the patient has an condition that prevents the procedure being performed at the bedside, such as obesity, goiter, or cervical spine diseases.

Regarding the technique used, some articles published during the 2003 SARS epidemic showed that, at the time, open tracheostomy was preferred to percutaneous tracheostomy (52). In open tracheostomies, the use of an electric scalpel should be avoided because of the potential theoretical risk of the presence of viruses in the smoke. When used, they must be associated with aspiration, as described above.

The procedure must be assisted by an anesthetist or intensivist, as there are needs for ventilatory maneuvers that the surgical team cannot control simultaneously, such as hyperpnea, complete pulmonary block, apnea, more distal or proximal positioning of the orotracheal tube in the trachea, and manipulation of the balloon. Finally, the ventilator expiratory valve settings must be controlled to ensure a pressure close to zero inside the tracheal lumen.

Before performing a tracheotomy, the tracheal cannula must already be attached to an obturator and filter as well as a syringe to inflate the balloon. The tracheal opening must be longitudinal and wide, preceded by the introduction of the orotracheal cannula so that the balloon is below the tracheal region to be manipulated. Reference points should not be passed in the trachea, to avoid encouraging attempts to reintroduce the cannula if it is inadvertently removed. In these cases, the orifice must be closed, and a new OTI must be performed by the anesthesia team.

The cannula must be fixed with stitches on the skin and its exchange must be postponed for at least 7 days or until the viral load is as low as possible (28,).

Since the creation of the surgical checklist by Haines and collaborators, in 2009 (56), it has been demonstrated that the use of this type of cheap and accessible technology reduces adverse events and increases safety for the patient, doctors, and other teams of the multidisciplinary team. Several authors have created specific safety checklists for tracheostomies in those infected by the virus (57,58). Among these, we highlight the checklist of the Society of Otorhinolaryngology in the United Kingdom, which has four stages, all starting with the letter “P” (59):

- Planning;
- Preparation;
- Performance;
- Post-Procedure.

Each stage includes specific safety checks, from general checks recommended by the World Health Organization to specific checks for tracheostomy. In the planning stage, before the day of surgery, aspects such as PPE, location to perform the tracheostomy, referral revision, separation of materials, and dedicated teams are finalized.

In the preparation stage, on the day of the procedure, checklist items include double checks of PPE and vestments (each team member checks the vestment of the other, as in moments preceding scuba dives or parachute jumps), materials, team readiness, planning the intraoperative steps, and preparing the cannula with couplings (as above).

The most critical stage is the performance of the procedure. The participation of the anesthetist or intensivist is crucial to guarantee total ventilatory paralysis and no risk of coughing, pre-oxygenation, opening of expiratory valves, clamping of the orotracheal tube, changing of the orotracheal tube position, opening of the trachea, replacement of the already prepared tracheal tube and immediate inflation of the balloon, ventilation without leakage, and confirmation of the position using capnography. At the end of the procedure, lack of clearance should be double checked, and team members who become contaminated during PPE removal should be alerted.

Finally, in the post-procedure stage, items include ensuring adequate manipulation of the patient (without risk of accidental loss of the tracheostomy), use of closed suction circuits, extended dressing manipulation or early cannula changing, and decannulation planning.

### Postoperative complications

The impact of COVID-19 on patients with head and neck cancer is relevant. A significant proportion of human resources and hospital services have experienced reduced demand, reducing the chances of early diagnosis of these cancers; thus, presentation of patients with tumors in more advanced stages is expected as the pandemic continues (60,61). Cancer patients show immunosuppression linked both to the cancer itself and to its treatment, which involves surgery, radiotherapy, chemotherapy, and immunotherapy. In addition, they must make frequent visits to the hospital, resulting in a greater exposure to and chance of developing the disease than for the general population, increasing their risk of SARS-CoV-2 infection, as shown in Wuhan (62).

During the COVID-19 pandemic, serious postoperative complications were observed in patients undergoing elective surgery. Chinese data from the beginning of the pandemic showed high rates of mortality and ICU admission. In a series involving 34 patients, all patients developed pneumonia, 15 required ICU admission, and seven died (63).

The highest rate of pulmonary complications and mortality from COVID-19 is in patients with the following characteristics: male, more than 70 years old, and with a history or current diagnosis of cancer and pneumopathy. Patients with head and neck cancer often have some, if not all, of these conditions: they usually have a history of smoking and drinking, impaired lung reserve, and greater susceptibility to infections, including aspiration pneumonia (64). Thus, the presence of fever and respiratory problems becomes a diagnostic challenge, as it is difficult to distinguish between pulmonary atelectasis and viral pneumonia (60).

### Non-surgical options and adjuvant therapy for cancer patients

A cancer patient has a-two times higher risk of SARS-CoV2 infection than does the general population because of immunosuppression caused by the tumor (35). However, malignant tumors in the head and neck are time-sensitive, and a delay in either diagnosis or treatment results in a worse prognosis. For example, with each month of delay in starting radiation therapy, the risk of death rises by 16% (34,66). In cases where monotherapy is indicated, as in early laryngeal tumors, because of the similar cure rate, one should choose the therapy that carries less risk of contamination for the patient, but this decision should be made by consensus between the medical team and the patient (62).

In more advanced tumors with locoregional dissemination, where, in addition to surgery, systemic treatment associated with radiotherapy must be performed, it is suggested to suspend the third cycle of cisplatin, except in cases where the toxicity was low in the first two cycles (62). In cases of COVID-19 infection, one must wait for the patient to recover and then start treatment as soon as possible (65).

As for the post-treatment follow-up, an individual assessment of each case must be carried out to determine whether a face-to-face consultation or telemedicine is appropriate (66).

### Multidisciplinary team support

During the pandemic, multidisciplinary support continues to be extremely important, especially regarding psychological assessment for anxiety that can be generated in relation to treatment and the chance of contamination by SARS-CoV-2 (35). Monitoring of nutritional status enables the determination of the best diet to avoid a significant weight loss during treatment, which could exacerbate immunosuppression (60).

### Effects of the pandemic on care

Most medical and hospital resources are being directed toward the treatment of COVID-19 patients. This, together with the need to reduce the exposure of patients and medical staff to the virus, has resulted in a reduction in the number of visits as a whole, and a reduction in the number of surgeries. In addition to chronic scarcity, channeling resources to COVID-19 also makes it difficult to apply best practices, especially in oncology, where treatments are often complex and costly.

It is estimated that approximately 50,000 Brazilians have missed being diagnosed with cancer during the pandemic, with a 30% drop in the demand to start treatment (67). At the Cancer Institute of the State of São Paulo, there was a 39% reduction in the number of first consultations in April 2020 when compared to that in April 2019. A similar pattern occurred with the total number of surgeries (55% reduction). This has also been observed in other cancer treatment centers in the city and state of São Paulo. This is expected to result in a greater volume of new cases in advanced stages, mainly because head and neck cancer is a clinically time-sensitive disease (35). It is estimated that the accumulation of cancer patients with delayed treatment or no treatment will lead to a 5% to 10% increase in mortality (68).

The build-up of cases will certainly compromise therapeutic decisions and prognoses. At the end of the acute phase of the pandemic, options for diagnosis and treatment will have to be adapted to the reality of resources, especially in a healthcare system with universal access, such as the Brazilian Unified Health System (SUS). The impact on survival and quality of life must be clearly presented to each patient. Teams should be trained to correctly indicate and provide palliative care to patients as an alternative treatment option. There must be no doubt in each case that this proposal is not based solely on economic grounds (69).

We cannot be unaware of this wave of patients who will seek assistance in the coming months. Each head and neck surgery service needs to estimate the probable accumulated volume of cases that will need to be treated, on the basis of the number of cases that were referred before the pandemic, in order to plan how to provide material and personnel resources to minimize losses and the risk of inadequate care.

### Teaching in medical residency

The COVID-19 pandemic has had an impact on head and neck surgery residency. The effects cannot be fully understood until the end of the pandemic. Medical residency programs, a postgraduate teaching modality with in-service training, have encountered a reduction in the number of surgical cases, for the reasons described above. There is currently a great deal of concern among medical residents in surgical fields regarding the impact on their training due to this decrease in the number of procedures.

In addition, the fight against COVID-19 has led to a drastic change in the medical residency program. On May 14, 2020, the Secretary of Higher Education in Brazil signed Technical Note No. 1/2020/CNRM/CGRS/DDES/SESU (70): recommendations regarding the development of activities for Medical Residency Programs (PRM) while coping with the COVID-19 pandemic (66). In that note, the National Medical Residency Commission (CNRM) considered the need for flexibility in PRM according to the level of epidemiological complexity, as determined by the Ministry of Health. It is up to the State Medical Residency Commission (CEREM) and the Medical Residency Committee (COREME) of each health institute to define the participation of the PRM in activities related to COVID-19.

In the state of São Paulo, where the pandemic was characterized as a Level 1 Emergency, the COREME of the Faculty of Medicine of the University of São Paulo (FMUSP) designated resident physicians of various specialties to act in the care of patients in the intensive care, emergency room, and nursing units of the Central Institute of the HCFMUSP. However, some residents continued to assist within their specialties, made possible by the other institutes belonging to the HCFMUSP. In the case of head and neck surgery, the Otávio Frias Filho Cancer Institute fulfilled this role. Even though the volume was reduced by half, residents maintained their activity in the specialty. Residents assigned to the Central Institute were only able to perform tracheostomies.

Within the PRM, the Technical Note stated that the maximum working hours of 60 hours per week should be maintained, comprising 80% to 90% practical assistance activities and 10% to 20% theoretical activities. Technology has enabled theoretical activities to be maintained, without the need to congregate in person, through clinical meetings and videoconferencing classes, in compliance with the recommendation “that they use messaging applications or other means of communication such as video classes.” The technical note emphasizes “that the theoretical workload should be set at the maximum allowed limit of 20% of the total workload, since in addition to the topics related to the respective medical specialties, it will be necessary to add those related to COVID-19, its complications, individual and collective protection strategies, *etc*. This results in the following distribution of the total workload: 48 hours/week in practical activities and 12 hours/week in theoretical activities.” It further states that “educational activities should be renegotiated, including the content on coping with COVID-19, within the scope of the PRM, addressing the teaching of the use of PPE and individual and collective protection measures, as well as the entire flow for care, treatment, and other matters pertinent to the broad training of resident physicians in facing the pandemic.”

A decision has not yet been made on the replacement of missed activities, although this issue was anticipated. (“The replacement of PRM activities, as originally conceived by the CNRM rules, which were unable to be performed in various scenarios during the pandemic, will be the object of analysis and subsequent decision by the CNRM once normality is resumed.”) Despite all of the difficulties and suffering, this health situation has imposed a restructuring of teaching formats, which will certainly be improved and incorporated into the regular course of the PRM.

### Final considerations

These recommendations are based on the experience of various services and medical societies, as well as recommendations from health authorities around the world, which have been adapted to the context of the Brazilian reality.

During the pandemic period, conduct must be adopted that prevents the spread of the disease, not only in the general population, but also among healthcare teams, as disease spread will harm the maintenance of the healthcare system. For doctors working in the cervico-oro-facial region, as well as dentists, the contamination risk is higher than for other specialists. Therefore, complete personal protection with an FFP2 mask (N95), gloves, waterproof aprons, goggles, and/or a facial visor must be used during outpatient care, in the operating room, and during the postoperative care of these patients.

Essential elective procedures should not be performed during this period, as they increase the risk of both contagion of the patient and complications in the postoperative period and may even lead to death. However, when there is a need to perform essential elective or elective urgency procedures, such as head and neck cancer surgeries, in addition to the use of complete PPE, the operation must be carried out in a controlled environment, with a trained team and the smallest possible number of participants. In this group of patients, neither surgical treatment, radiotherapy, nor systemic therapies should be postponed because of the time-sensitive nature of head and neck cancers.

## AUTHOR CONTRIBUTIONS

Kulcsar MAV was responsible for data curation and manuscript drafting, editing and review. Montenegro FLM, Santos ABO, Tavares MR and Arap SS were responsible for the manuscript writing, editing and review. Kowalski LP was responsible for the study supervision and manuscript writing, editing and review.

## References

[1] World Health Organization (16 April 2020). WHO Director-General's opening remarks at the Mission briefing on COVID-19.

[2] World Health Organization Rolling updates on coronavirus disease (COVID-19).

[3] Ministério da Saúde Brasil confirma primeiro caso da doença.

[4] Ministério da Saúde Portal do COVID-19.

[5] Setti L, Passarini F, De Gennaro G, Barbieri P, Perrone MG, Borelli M (2020). Airborne Transmission Route of COVID-19: Why 2 Meters/6 Feet of Inter-Personal Distance Could Not Be Enough. Int J Environ Res Public Health.

[6] van Doremalen N, Bushmaker T, Morris DH, Holbrook MG, Gamble A, Williamson BN (2020). Aerosol and Surface Stability of SARS-CoV-2 as Compared with SARS-CoV-1. N Engl J Med.

[7] Deutsche Welle (DW) Os números sobre a pandemia de coronavírus.

[8] Anastassopoulou C, Russo L, Tsakris A, Siettos C (2020). Data-based analysis, modelling and forecasting of the COVID-19 outbreak. PLoS One.

[9] Sit THC, Brackman CJ, Ip SM, Tam KWS, Law PYT, To EMW (2020). Infection of dogs with SARS-CoV-2. Nature.

[10] Huang C, Wang Y, Li X, Ren L, Zhao J, Hu Y (2020). Clinical features of patients infected with 2019 novel coronavirus in Wuhan, China. Lancet.

[11] Lechien JR, Chiesa-Estomba CM, De Siati DR, Horoi M, Le Bon SD, Rodriguez A (2020). Olfactory and gustatory dysfunctions as a clinical presentation of mild-to-moderate forms of the coronavirus disease (COVID-19): a multicenter European study. Eur Arch Otorhinolaryngol.

[12] Buscarini E, Manfredi G, Brambilla G, Menozzi F, Londoni C, Alicante S (2020). Gastrointestinal symptoms as COVID-19 onset in hospitalized Italian patients. medRxiv.

[13] Chan JF, Zhang AJ, Yuan S, Poon VK, Chan CC, Lee AC (2020). Simulation of the clinical and pathological manifestations of Coronavirus Disease 2019 (COVID-19) in golden Syrian hamster model: implications for disease pathogenesis and transmissibility. Clin Infect Dis.

[14] Zhou F, Yu T, Du R, Fan G, Liu Y, Liu Z (2020). Clinical course and risk factors for mortality of adult inpatients with COVID-19 in Wuhan, China: a retrospective cohort. Lancet.

[15] Guo YR, Cao QD, Hong ZS, Tan YY, Chen SD, Jin H J (2020). The origin, transmission and clinical therapies on coronavirus disease 2019 (COVID-19) outbreak - an update on the status. Mil Med Res.

[16] Wu Z, McGoogan JM (2020). Characteristics of and Important Lessons From the Coronavirus Disease 2019 (COVID-19) Outbreak in China: Summary of a Report of 72314 Cases From the Chinese Center for Disease Control and Prevention. JAMA.

[17] Anelli F, Leoni G, Monaco R, Nume C, Rossi RC, Marinoni G (2020). Italian doctors call for protecting healthcare workers and boosting community surveillance during covid-19 outbreak. BMJ.

[18] Jornal O Globo Sociedade Brasil registrou 31,7 mil profissionais de saúde infectados pela Covid-19.

[19] Wang D, Hu B, Hu C, Zhu F, Liu X, Zhang J (2020). Clinical Characteristics of 138 Hospitalized Patients With 2019 Novel Coronavirus-Infected Pneumonia in Wuhan, China. JAMA.

[20] Kulcsar MA, Montenegro FL, Arap SS, Tavares MR, Kowalski LP (2020). High Risk of COVID-19 Infection for Head and Neck Surgeons. Int Arch Otorhinolaryngol.

[21] Kowalski LP, Sanabria A, Ridge JA, Ng WT, de Bree R, Rinaldo A (2020). COVID-19 pandemic: Effects and evidence-based recommendations for otolaryngology and head and neck surgery practice. Head Neck.

[22] Ng K, Poon BH, Kiat Puar TH, Shan Quah JL, Loh WJ, Wong YJ (2020). COVID-19 and the Risk to Health Care Workers: A Case Report. Ann Intern Med.

[23] Conselho Federal de Enfermagem Enfermagem realiza ato histórico em homenagem aos mortos pela COVID-19.

[24] ENT UK Guidance for ENT during the COVID-19 pandemic.

[25] Conselho Regional de Medicina do Estado de São Paulo - CREMESP Notícias. Manejo clínico do COVID-19.

[26] Setzen G, Anne S, Brown III EG, Denneny III JC, Dubin MG, Ishman SL Guidance for Return to Practice for Otolaryngology-Head and Neck Surgery Part One. 2020. American Academy of Otolaryngology - Head and Neck Surgery.

[27] Associação Brasileira de Otorrinolaringologia e Cirurgia Cérvico-Facial (2020). 1a nota de orientação aos médicos otorrinolaringologistas em relação è doença causada pelo novo coronavírus (COVID-19) - 03 de março de.

[28] Sociedade Brasileira de Cirurgia Torácica https://www.sbct.org.br/recomendacoes-da-sociedade-brasileira-de-cirurgia-toracica-sbct-para-realizacao-de-traqueostomias-e-manejo-da-via-aerea-em-casos-suspeitos-ou-confirmados-de-infeccao-pelo-novo-coronavirus-c/.

[29] Day AT, Sher DJ, Lee RC, Truelson JM, Myers LL, Sumer BD (2020). Head and neck oncology during the COVID-19 pandemic: Reconsidering traditional treatment paradigms in light of new surgical and other multilevel risks. Oral Oncol.

[30] Conselho Federal de Medicina Combate è COVID-19: CFM divulga orientações para o trabalho dos médicos durante o período de enfrentamento do coronavírus.

[31] Centers for Medicare & Medicaid Services - CMS Releases Recommendations on Adult Elective Surgeries, Non-Essential Medical, Surgical, and Dental Procedures During COVID-19 Response.

[32] American College of Surgeons COVID 19: Considerations for Optimum Surgeon Protection.

[33] Fakhry N, Schultz P, Moriniàre S, Breuskin I, Bozec A, Vergez S (2020). French consensus on management of head and neck cancer surgery during COVID-19 pandemic. Eur Ann Otorhinolaryngol Head Neck Dis.

[34] Wu V, Noel CW, Forner D, Zhang ZJ, Higgins KM, Enepekides DJ (2020). Considerations for head and neck oncology practices during the coronavirus disease 2019 (COVID-19) pandemic: Wuhan and Toronto experience. Head Neck.

[35] Al Shamsi HO, Alhazzani W, Alhuraiji A, Coomes EA, Chemaly RF, Almuhanna M (2020). A Practical Approach to the Management of Cancer Patients During the Novel Coronavirus Disease 2019 (COVID-19) Pandemic: An International Collaborative. Oncologist.

[36] Chow VLY, Chan JYW, Ho VWY, Lee GCC, Wong MMK, Wong STS (2020). Conservation of personal protective equipment for head and neck cancer surgery during COVID-19 pandemic. Head Neck.

[37] Givi B, Schiff BA, Chinn SB, Clayburgh D, Iyer NG, Jalisi S (2020). Safety Recommendations for Evaluation and Surgery of the Head and Neck During the COVID-19 Pandemic. JAMA Otolaryngol Head Neck Surg.

[38] Mason DJ, Friese CR (2020). Protecting health care workers against COVID-19—and being prepared for future pandemics. JAMA Health Forum.

[39] American College of Surgeons COVID-19: Guidance for Triage of Non-Emergent Surgical Procedures.

[40] Organização Panamericana da Saúde Prevenção e controle de infecção durante os cuidados de saúde quando houver suspeita de infecção pelo novo coronavírus (nCoV): diretrizes provisórias.

[41] Baud G, Brunaud L, Lifante JC, Tresallet C, Sebag F, Bizard JP (2020). Chirurgie endocrinienne au cours et au décours de l’épidémie de COVID-19: Recommandations de l’AFCE [Endocrine surgery during and after the COVID-19 epidemic: guidelines from AFCE].

[42] Prachand VN, Milner R, Angelos P, Posner MC, Fung JJ, Agrawal N (2020). Medically Necessary, Time-Sensitive Procedures: Scoring System to Ethically and Efficiently Manage Resource Scarcity and Provider Risk During the COVID-19 Pandemic. J Am Coll Surg.

[43] Agência Nacional de Vigilância Sanitária - ANVISA Notas técnicas da Anvisa.

[44] Colégio Brasileiro de Cirurgiões- CBC Sociedade Brasileira de Cirurgia Oncológica - SBCO. Sociedade Brasileira de Ortopedia e Traumatologia - SBOT. Nota de esclarecimento sobre o COVID-19.

[45] American Head and Neck Society Endocrine Surgery during the COVID-19 pandemic.

[46] Chen AY, Shindo M (2020). Ethical framework for head and neck endocrine surgery in the COVID-19 pandemic. Head Neck.

[47] Guimarães HP, Schubert DUC, Rodrigues RR, Freitas APR, Corrêa TD, de Araujo K Recomendações para Intubação Orotraqueal em pacientes portadores de COVID-19 - Versão N.3/2020. Associação Brasileira de Medicina de emergência.

[48] Bhatraju PK, Ghassemieh BJ, Nichols M, Kim R, Jerome KR, Nalla AK (2020). Covid-19 in Critically Ill Patients in the Seattle Region - Case Series. N Engl J Med.

[49] McGrath BA, Brenner MJ, Warrillow SJ, Pandian V, Arora A, Cameron TS (2020). Tracheostomy in the COVID-19 era: global and multidisciplinary guidance. Lancet Respir Med.

[50] Liu CC, Rudmik L (2016). A Cost-effectiveness Analysis of Early vs Late Tracheostomy. JAMA Otolaryngol Head Neck Surg.

[51] Adly A, Youssef TA, El-Begermy MM, Younis HM (2018). Timing of tracheostomy in patients with prolonged endotracheal intubation: a systematic review. Eur Arch Otorhinolaryngol.

[52] Tien HC, Chughtai T, Jogeklar A, Cooper AB, Brenneman F (2005). Elective and emergency surgery in patients with severe acute respiratory syndrome (SARS). Can J Surg.

[53] Sociedade Brasileira de Cirurgia de Cabeça e Pescoço Recomendação da SBCCP para traqueostomias e manejo da via aérea em casos suspeitos ou confirmados de COVID-19. (18 de março de 2020).

[54] Patel ZM, Fernandez-Miranda J, Hwang PH, Nayak JV, Dodd R, Sajjadi H (2020). Letter: Precautions for Endoscopic Transnasal Skull Base Surgery During the COVID-19 Pandemic. Neurosurgery.

[55] Tay JK, Khoo ML, Loh WS (2020). Surgical Considerations for Tracheostomy During the COVID-19 Pandemic: Lessons Learned From the Severe Acute Respiratory Syndrome. JAMA Otolaryngol Head Neck Surg.

[56] Haynes AB, Weiser TG, Berry WR, Lipsitz SR, Breizat AH, Dellinger EP (2009). A surgical safety checklist to reduce morbidity and mortality in a global population. N Engl J Med.

[57] Broderick D, Kyzas P, Sanders K, Sawyerr A, Katre C, Vassiliou L (2020). Surgical tracheostomies in Covid-19 patients: important considerations and the “5Ts” of safety. Br J Oral Maxillofac Surg.

[58] Dharmarajan H, Snyderman CH (2020). Tracheostomy time-out: New safety tool in the setting of COVID-19. Head Neck.

[59] Jacob T, Walker A, Mantelakis A, Gibbins N, Keane O (2020). A framework for open tracheostomy in COVID-19 patients. Clin Otolaryngol.

[60] Aminian A, Safari S, Razeghian-Jahromi A, Ghorbani M, Delaney CP (2020). COVID-19 Outbreak and Surgical Practice: Unexpected Fatality in Perioperative Period. Ann Surg.

[61] Topf MC, Shenson JA, Holsinger FC, Wald SH, Cianfichi LJ, Rosenthal EL (2020). Framework for prioritizing head and neck surgery during the COVID-19 pandemic. Head Neck.

[62] Liang W, Guan W, Chen R, Wang W, Li J, Xu K (2020). Cancer patients in SARS-CoV-2 infection: a nationwide analysis in China. Lancet Oncol.

[63] Lei S, Jiang F, Su W, Chen C, Chen J, Mei W (2020). Clinical characteristics and outcomes of patients undergoing surgeries during the incubation period of COVID-19 infection. EClinicalMedicine.

[64] Silverman DA, Lin C, Tamaki A, Puram SV, Carrau RL, Seim NB (2020). Respiratory and pulmonary complications in head and neck cancer patients: Evidence-based review for the COVID-19 era. Head Neck.

[65] Hanna TP, Evans GA, Booth CM (2020). Cancer, COVID-19 and the precautionary principle: prioritizing treatment during a global pandemic. Nat Rev Clin Oncol.

[66] Chaves ALF, Castro AF, Marta GN, Junior GC, Ferris RL, Giglio RE (2020). Emergency changes in international guidelines on treatment for head and neck cancer patients during the COVID-19 pandemic. Oral Oncol.

[67] Jornal “O Estado de São Paulo” Pandemia do coronavirus faz ao menos 50 mil brasileiros deixaram de ser diagnosticados com câncer.

[68] The Lancet Oncology (2020). Safeguarding cancer care in a post-COVID-19 world. Lancet Oncol.

[69] Porta C (2020). Editorial debate: Challenges an oncologist has to face during the SARS-CoV-2 pandemic within a universal healthcare system. Version 2. ESMO Open.

[70] Ministério da Educação Nota Técnica N° 1/2020/CNRM/CGRS/DDES/SESU/SESU.

